# Self-efficacy and positive thinking as predictors of health-related quality of life in women with stress urinary incontinence

**DOI:** 10.1186/s12905-022-02025-0

**Published:** 2022-11-11

**Authors:** Ching Hui Chien, Xuan Yi Huang, Shu Pen Hsu, Yi Hua Yen, Hun Shan Pan, Feng Chu Yen

**Affiliations:** 1 College of Nursing, National Taipei University of Nursing and Healh Sciences, No.365, Ming-te Road, Peitou District, Taipei City, 112 Taiwan; 2 Department of Nursing, Shin Kong Wu Ho-Su Memorial Hospital, Taipei City, Taiwan; 3 Physiology Examination Room, Shin Kong Wu Ho-Su Memorial Hospital, Taipei City, Taiwan; 4 Department of Obstetrics and Gynecology, Shin Kong Wu Ho-Su Memorial Hospital, Taipei City, Taiwan; 5 College of Medicine, Fu Zen Catholic University, New Taipei City, Taiwan; 6 Department of Nursing, National Taiwan University Hospital, Taipei City, Taiwan

**Keywords:** Stress urinary incontinence, Positive thinking, Self-efficacy, Quality of life

## Abstract

**Background:**

Stress urinary incontinence (SUI), which causes involuntarily leakage of urine, has an impact on many women and may affect self-efficacy, which, in turn, can lead to poor health-related quality of life (QOL). This study aimed to explore the effects of sociodemographic and health information, symptom distress, self-efficacy, and positive thinking on the health-related QOL (general QOL and urinary incontinence-specific QOL) of women with SUI.

**Methods:**

A cross-sectional study design was used. Women with SUI were recruited from the obstetrics and gynecology outpatient department and urodynamics examination room of a hospital by convenience sampling from August 2021 to March 2022. Participants were surveyed on the following questionnaires: Urogenital Distress Inventory, Geriatric Self-efficacy Index for Urinary Incontinence, Positive Thinking Scale, 12-Item Short-Form Health Survey (SF-12), and Incontinence Impact Questionnaire Short Form.

**Results:**

Participants (*N* = 135) had a mean age of 53.76 years old. The mean SF-12 physical component summary score was 48.48 (physical QOL), and the mental component summary score was 46.56 (mental QOL). The urinary incontinence-specific QOL score was 16.01. Women with greater positive thinking and higher self-efficacy for urinary incontinence had better physical and mental QOL. Women with less symptom distress of urinary incontinence and higher self-efficacy for urinary incontinence had better urinary incontinence-specific QOL.

**Conclusion:**

The health-related QOL of women with SUI is affected by many factors, including positive thinking, self-efficacy, and symptom distress. Healthcare professionals can provide multifaceted programs to improve the health-related QOL of women with SUI.

## Highlights

• General quality of life was influenced by positive thinking in women with SUI.

• Health-related QOL was influenced by self-efficacy in women with SUI.

• Symptom distress was a predictor of urinary incontinence-specific QOL.

• Multi-element programs are needed to improve the health-related QOL of women with SUI.

## Background

Urinary incontinence refers to the involuntary outflow of urine from the body of an individual. Stress urinary incontinence (SUI) occurs when the detrusor muscles do not contract during instances of increased abdominal pressure, caused by activities such as coughing, laughing, lifting weights, and sneezing [1]. A survey showed that 45.9% of women have experienced SUI [2]. SUI can be alleviated through weight loss, pelvic floor muscle training (PFMT), extracorporeal electromagnetic stimulation, medications, and surgery [1, 3, 4]. Some women consider urinary incontinence to be a normal phenomenon caused by aging or other reasons, and, thus, they are not properly evaluated and treated [5]. In more conservative eastern countries, in particular, many women seek medical attention only when the distress caused by SUI becomes severe, and many women with mild SUI do not receive any treatment [5, 6].

Symptoms of urinary incontinence and the distress generated can leave women feeling alone [7]. Many women with urinary incontinence are self-deprecating, shy, remorseful, and embarrassed and feel hopeless [8]; they withdraw socially or avoid certain activities [8, 9], thereby worsening their health-related quality of life (QOL) [6, 10]. Evidence has shown that the Coronavirus Disease 2019 (COVID-19) has had a negative impact on individuals’ mental health, which may have resulted in the deterioration of health-related QOL in women with SUI [11].

QOL is a subjective, multifaceted, and complex concept and is one of the vital indicators used when evaluating the benefits of care [12, 13]. Scholars argue that an individual’s health-related QOL consists of different levels, including physiological, psychological, social, and spiritual and involves disease- and treatment-related symptoms [13]. Health-related QOL can be assessed through disease-specific and general approaches [12, 13].

Urinary incontinence increases the incidence of social dysfunction in women [9]. Women with more symptom distress of urinary incontinence experience a greater impact of SUI on their travel, social and physical activities, and emotional well-being [6]. A study in Australia found that, when patients with urinary incontinence had mixed symptoms of depression, all aspects of their health-related QOL were worse than those of other patients without symptoms of depression [10].

Self-efficacy is a manifestation of an individual’s confidence in achieving a certain goal [14, 15]. Self-efficacy for urinary incontinence refers to an individual’s confidence or self-efficacy in preventing involuntary urinary leakage [16]. Self-efficacy determines whether an individual can adopt or modify self-care behavior after obtaining relevant care information and knowledge [14, 15]. Women who have a higher self-efficacy for urinary incontinence are expected to be more able to adopt or modify self-care behaviors to prevent involuntary urinary leakage [14, 16], thereby maintaining their health-related QOL [16, 17]. If the self-efficacy for urinary incontinence in women with SUI can be improved, then their health-related QOL can be enhanced [4, 16].

Positive thinking is both a positive cognition and a coping behavior [18]. Previous studies have shown that individuals with greater positive thinking have higher life satisfaction [19] and are happier [20]. According to the author’s best knowledge, no studies have explored the correlation between positive thinking and health-related QOL (general QOL and urinary incontinence-specific QOL) in women with SUI. Therefore, this study aimed to explore health-related QOL and its predictors in women with SUI. The factors associated with health-related QOL explored in this study include sociodemographic and health characteristics, symptom distress, self-efficacy for urinary incontinence, and positive thinking.

## Methods

### Design and study sample

A cross-sectional study design was used. Women with SUI were recruited from the obstetrics and gynecology outpatient department and urodynamics examination room of a hospital through convenience sampling from August 2021 to March 2022. During the study period, potential participants were referred by an obstetrician. In addition, flyers used to advertise the study were placed in the outpatient department to recruit participants from the hospital. Women who were willing to participate in the study could contact the investigator. The investigator ensured that women met the inclusion criteria by interviewing them and checking their medical records. The research methodology, content, and study purpose were explained to eligible women. After obtaining verbal and written consent, a structured questionnaire was given to participants. The inclusion criteria were as follows: women with SUI confirmed by a physician; over 20 years old; and agreed to participate in the study and sign a consent form. Exclusion criteria were as follows: women with a Barthel Index score below 90; those who had urinary catheters; those who were unable to communicate; those with urinary incontinence due to cancer or severe illness; and those with psychiatric disorders, such as major depression and dementia.

### Estimated sample size

A correlation coefficient (*r*) of 0.70 between the score on the Geriatric Self-efficacy Index for Urinary Incontinence (GSE-UI) and the score on the Incontinence QOL questionnaire previously reported in patients in an incontinence study was used as the basis for sample size estimation [16]. The sample size was estimated using G*Power Version 3.1.9.2 [21] with the following parameters: for linear regression statistical analysis, *α* = 0.05, power = 80%, and independent variables = 14. This resulted in a required sample size of at least 33 participants. Of 174 potential participants, 32 did not meet the inclusion criteria, and seven declined to participate due to the time commitment required. A total of 135 participants completed the questionnaire.

### Measurements

#### Sociodemographic and health information list

The data collected on this scale consisted of basic demographic properties and disease treatment characteristics. The items included age, education level, religious beliefs, marital status, occupation, self-reported financial status, chronic disease, self-reported health status, menopausal status (e.g., perimenopausal, post-menopausal), and SUI treatment. In addition, we recorded the numbers of newly diagnosed COVID-19 cases reported by the Central Epidemic Command Center in Taiwan [22] on the dates when participants completed the questionnaire.

#### Distress caused by symptoms of urinary incontinence

A short version of the Urogenital Distress Inventory (UDI) included a total of six questions, which were part of the three subscales of obstruction, stress, and irritation. A 4-point scoring method was adopted, with a score range of 0–3, and the score was linearly scaled to 0–100. A higher score indicates greater distress [23, 24]. The scale has acceptable reliability and validity [23]. A UDI-6 score of 33.33 has a specificity of 83.3% and a sensitivity of 97.0% for detecting greater distress caused by urinary incontinence [25]. The Chinese version scale has acceptable criterion, convergent, and discriminant validity [6, 26] as well as acceptable reliability, with a Cronbach’s *α* of 0.80 [26]. The Cronbach’s *α* in this study was 0.75.

#### Self-efficacy index for urinary incontinence

A total of 12 questions in the GSE-UI were administered, using a 10-point scoring method. The total score ranged from 0 to 120. A higher score indicates better self-efficacy for urinary incontinence [16, 27]. This original English scale has a specificity of 78.2% and a sensitivity of 75.1% for detecting clinically significant changes in patients with urinary incontinence. The Cronbach’s *α* of the internal consistency of the scale was 0.90 [27]. With the consent of the original scale developers, this study used the forward-backward method to translate the English version scale into a traditional Chinese version scale [28]. Then, expert validity and face validity were verified, and the contents of the scale were adjusted according to expert opinion to construct a scale that could be used with women with SUI in Taiwan. The content validity index was 1.0 [29], and the Cronbach’s *α* was 0.92.

#### Positive thinking

The Positive Thinking Scale comprises personal satisfaction and goal pursuit. The scale includes a total of 18 questions and uses a 5-point scoring method. The total score ranges from 18 to 90. A higher score indicates a greater perception of positive thinking [18, 30, 31]. The Korean version of the original scale has acceptable discriminant and convergence validity. The Cronbach’s *α* of the total scale internal consistency was 0.88 [18, 19]. The Chinese version scale has acceptable concurrent, predictive, and construct validity, and the Cronbach’s *α* of the internal consistency ranged from 0.88 to 0.98 [30, 31]. In this study, the Cronbach’s *α* was 0.93.

#### General quality of life

The Chinese version of the 12-Item Short-Form Health Survey (SF-12) was used to assess general QOL in the study participants. The SF-12 is a short version of the SF-36, easy to complete, and less burdensome [32]. The SF-12 consists of eight domains, namely, general health, physical functioning, bodily pain, vitality, role physical, role emotional, social functioning, and mental health. The scores are converted into a physical component summary (PCS) score and mental component summary (MCS) score by using PRO CoRE software. A higher score reflects better physical and mental QOL. The SF-12 can explain more than 90% of the score variation of the SF-36 with acceptable reliability [33, 34]. The Chinese version of the SF-12 has acceptable reliability and validity [32].

#### Urinary incontinence-specific quality of life

The Chinese version of the Incontinence Impact Questionnaire short form (IIQ-7) has been used widely to assess urinary incontinence-specific QOL [26]. This study used the IIQ-7 to assess urinary incontinence-specific QOL in women with SUI. The scale contains seven questions that cover four subscales, namely, travel, physical activity, emotional health, and social/relationships. Using a 4-point scoring method, the score was linearly scaled to 0–100. A higher score indicates a greater impact caused by urinary incontinence and worse urinary incontinence-specific QOL [24, 26]. The Chinese version of IIQ-7 has criterion, convergent, and discriminant validity. The Cronbach’s *α* of the total scale reliability was 0.93 [26]. The Cronbach’s *α* in this study was 0.89.

### Ethical considerations

This study was approved by the Institutional Review Board of the hospital (No. 20210107R) and was performed in accordance with the Declaration of Helsinki. The researchers explained the content and purpose of the study to women who met the inclusion criteria and gave them time to reflect on their willingness to participate in the study. Questionnaires were completed only after women had agreed and signed the research consent form. Participants were allowed to discontinue or withdraw from this study at any time without prejudice to their therapeutic rights. The questionnaire information was coded in lieu of participant names and was locked in a steel cabinet in the investigator’s office to protect the data and ensure subject privacy.

### Statistical analysis

Statistical analysis was performed using IBM SPSS Statistics version 20.0. Descriptive statistical methods included mean, standard deviation (SD), frequency, percentage, percentile, and range. Inferential statistics consisted of independent sample *t*-tests, one-way analysis of variance, Pearson product-difference correlations, and multiple linear regressions. When using stepwise regression for multivariate analysis, the variants were included in the analysis along with the statistically significant variables of univariate analysis, which included urinary incontinence symptom distress, urinary incontinence self-efficacy, and positive thinking. A two-tailed test was used, and a *p*-value of less than 0.05 indicated statistical significance.

## Results

A total of 135 women were enrolled in this study. The mean age was 53.76 (SD = 12.37) years. Of the participants, 80.0% had a high school education or above, 90.4% were married, 73.3% had religious beliefs, 52.6% were unemployed or retired, and 51.9% perceived their financial status as sufficient. Further, 60.7% had chronic diseases, 46.7% were post-menopausal, 24.4% were in perimenopause, 24.4% of women with urinary incontinence performed PFMT, and 29.6% received extracorporeal magnetic innervation (ExMI) (Table 1).

**Table 1:** Sociodemographic and health characteristics of participants (*N* = 135)

Variable	*n*	*%* [Mean ± SD]
Age		[53.76 ± 12.37] Range = 25 to 82 Interquartile range [IQR] Q1 = 43; Q2 = 52; Q3 = 64; Q4 = 82
Education level		
Elementary school and below	27	20.0
Junior and senior high school	56	41.5
College and above	52	38.5
Marital status		
Single/divorced	13	9.6
Married/cohabiting	122	90.4
Religious beliefs		
None	36	26.7
Yes	99	73.3
Occupation		
None/retired	71	52.6
Employment	64	47.4
Perceived financial status		
Insufficient	30	22.2
Balanced	35	25.9
Sufficient	70	51.9
Chronic disease		
No	53	39.3
Yes	82	60.7
Perceived physical health		[8.41 ± 1.54]
Perimenopausal		
No	102	75.6
Yes	33	24.4
Post-menopausal		
No	72	53.3
Yes	63	46.7
Treatment for urinary incontinence		
Medication	14	10.4
Surgery	23	17.0
Pelvic floor muscle training	33	24.4
Extracorporeal magnetic innervation	40	29.6
Newly diagnosed COVID-19 cases		[45.70 ± 82.01] Range = 0 to 630 Interquartile range [IQR] Q1 = 8; Q2 = 16; Q3 = 64; Q4 = 630

### Predictors of general quality of life

The mean PCS score was 48.48 (SD = 7.54). In the univariate analysis, for women with SUI, there were significant correlations between age, education level, occupation, chronic disease, menopausal status (perimenopausal and post-menopausal), perceived physical health, symptom distress, self-efficacy, and positive thinking with PCS scores. Further stepwise regression analysis found that the most important factors that predicted PCS scores in women with SUI were as follows: perceived physical health (*B* = 1.35, *p* < .001), chronic diseases (*B* = − 3.19, *p* = .006), self-efficacy (*B* = 0.04, *p* = .034), perimenopause (*B* = − 3.11, *p* = .016), positive thinking (*B* = 0.13, *p* = .016), and post-menopause (*B* = − 2.43, *p* = .035; No > Yes). These factors explain 38% of the variance in PCS scores. That is, women with better perceived physical health and higher self-efficacy for urinary incontinence and women with a greater perception of positive thinking experienced better physical QOL. Patients without a chronic disease had better physical QOL than did those with chronic diseases. Compared with women who were perimenopausal or post-menopausal, those who were not in perimenopause or post-menopause had better physical QOL (Tables 2, 3 and 4).

**Table 2:** Correlations between sociodemographic and health characteristics and health-related quality of life (*N* = 135)

	General quality of life				Urinary incontinence-specific quality of life	
	Physical component summary		Mental component summary			
Variable	Mean (SD)	*t/F*	Mean (SD)	*t/F*	Mean (SD)	*t/F*
Education level		4.25*		0.47		3.17*
1. Elementary school and below	46.24 (9.35)	3 > 1	46.37 (12.79)		22.35 (18.36)	1 > 3
2. Junior and senior high school	47.46 (6.71)		47.49 (10.59)		13.20 (14.86)	
3. College and above	50.74 (6.87)		45.65 (7.00)		15.73 (14.69)	
Religious beliefs		.81		.08		−2.89**
None	49.35 (7.13)		46.67 (5.47)		10.42 (12.35)	
Yes	48.16 (7.69)		46.51 (11.05)		18.04 (16.46)	
Marital status		−.53		.44		−2.57*
Single/divorced	47.43 (8.24)		47.71 (12.29)		9.89 (7.87)	
Married/cohabiting	48.59 (7.49)		46.43 (9.61)		16.66 (16.30)	
Occupation		−3.67***		.91		2.17*
None/Retired	46.32 (8.25)		47.29 (11.00)		18.76 (16.86)	
Employment	50.88 (5.85)		45.74 (8.41)		12.95 (14.04)	
Perceived financial status		.86		.89		.76
Insufficient	46.99 (7.80)		46.07 (13.15)		18.21 (17.00)	
Balanced	49.39 (7.71)		44.93 (9.09)		13.47 (15.95)	
Sufficient	48.67 (7.36)		47.58 (8.52)		16.33 (15.24)	
Chronic disease		4.02***		.19		−0.98
No	51.56 (5.83)		46.76 (8.49)		14.35 (14.85)	
Yes	46.49 (7.88)		46.43 (10.69)		17.07 (16.38)	
Perimenopausal		2.40*		0.68		−0.77
No	49.35 (7.53)		46.88 (9.78)		15.41 (16.36)	
Yes	45.79 (7.00)		45.54 (10.16)		17.86 (13.98)	
Post-menopausal		4.11***		−1.60		−2.24*
No	50.84 (6.18)		45.26 (7.75)		13.19 (14.32)	
Yes	45.79 (8.08)		48.03 (11.70)		19.22 (16.88)	

*p < .05, **p < .01, ***p < .001

**Table 3:** Pearson correlation coefficients (*N* = 135)

		General quality of life		
		Physical component summary	Mental component summary	Urinary incontinence-specific quality of life
Variable	Mean (SD)	*r*	*r*	*r*
Age	53.76 (12.37)	−.30***	.14	.23**
Perceived physical health	8.41 (1.54)	.43***	.26***	−.19*
Number of newly diagnosed COVID-19 cases	45.70 (82.01)	.14	.09	−0.17
Urinary incontinence symptom distress	22.78 (15.36)	−.21*	−.15	.73***
Urinary incontinence self-efficacy	73.60 (27.33)	.32***	.22**	−.53***
Positive thinking	68.69 (10.74)	.30***	.60***	−.11
Mean (SD)		48.48 (7.54)	46.56 (9.85)	16.01 (15.80)

*p < .05, **p < .01, ***p < .001

**Table 4:** Predictors of health-related quality of life (*N* = 135)

Variable	*B*	*SE*	*t*	*p-*value	*R*^2^	△*R*^2^	*F*	*p-*value
Physical component summary								
(Intercept)	28.83	4.15						
Perceived physical health	1.35	0.38	3.59	<.001	.18	.18	29.67	<.001
Chronic disease	−3.19	1.14	−2.81	.006	.26	.08	23.05	<.001
Urinary incontinence self-efficacy	0.04	0.02	2.14	.034	.31	.05	19.78	<.001
Perimenopausal	−3.11	1.27	−2.45	.016	.34	.02	16.48	<.001
Positive thinking	0.13	0.05	2.44	.016	.36	.03	14.61	<.001
Post-menopausal	−2.43	1.14	−2.13	.035	.38	.02	13.27	<.001
Mental component summary								
(Intercept)	5.63	4.54						
Positive thinking	0.54	0.06	8.52	<.001	.36	.36	76.00	<.001
Urinary incontinence self-efficacy	0.06	0.02	2.24	.027	.39	.03	41.66	<.001
Urinary incontinence-specific quality of life								
(Intercept)	9.62	4.07						
Urinary incontinence Symptoms distress	0.65	0.07	9.28	<.001	.54	.54	155.20	<.001
Urinary incontinence self-efficacy	−0.11	0.04	−2.90	.004	.57	.03	86.10	<.001

The mean MCS score was 46.56 (SD = 9.85). Univariate analysis revealed significant correlations between perceived physical health, self-efficacy, and positive thinking with MCS scores in women with SUI. Further stepwise regression analysis found that positive thinking (*B* = 0.54, *p* < .001) and self-efficacy (*B* = 0.06, *p* = 0.027) were the most important factors that predict MCS scores in women with SUI and explained 39% of the variance in MCS scores. That is, women with more positive thinking and higher self-efficacy for urinary incontinence experienced better mental QOL (Tables 2, 3 and 4).

### Predictors of urinary incontinence-specific quality of life

The mean score of the urinary incontinence-specific QOL was 16.01 (SD = 15.80). Univariate analysis found significant correlations between age, education, religious beliefs, marital status, occupation, post-menopausal status, perceived physical health, symptom distress, and self-efficacy with urinary incontinence-specific QOL scores in women with SUI. Further stepwise regression analysis found that symptom distress (*B* = 0.65, *p* < .001) and self-efficacy (*B* = − 0.11, *p* = .004) were the most important factors for predicting the scores of urinary incontinence-specific QOL of women with SUI and explained 57% of the variance in urinary incontinence-specific QOL scores. That is, women with less symptom distress of urinary incontinence and higher self-efficacy for urinary incontinence had a better urinary incontinence-specific QOL (Tables 2, 3 and 4).

## Discussion

This study is the first to explore the effect of positive thinking on health-related QOL among women with SUI. The results showed that the physical health information of women with SUI included essential factors that significantly affected their physical QOL, namely, chronic disease, menopausal status, and self-reported health status. Moreover, women with greater positive thinking experienced better physical and mental QOL. Women with higher self-efficacy for urinary incontinence had better health-related QOL. Women with less symptom distress of urinary incontinence had better urinary incontinence-specific QOL.

In the current study, the results indicated that women with SUI had mean PCS and MCS scores of 48.48 and 46.56, respectively, during the COVID-19 pandemic. These scores are significantly better than those obtained in a study conducted in Bangladesh [35]. In that study, the data of 126 women with different types of urinary incontinence showed that these women had PCS and MCS scores of 33.9 and 42.0, respectively. One potential reason for the difference in scores between the two studies was that the current study focused on women with SUI and excluded women diagnosed with major depression. In addition, the PCS score of women with SUI in the current study was similar to or better than that of the Taiwanese female spouses of patients with prostate cancer (between 45.48 and 48.03), but their MCS score was worse (between 52.28 and 53.37) [36]. This difference could be attributed to the effect of SUI on women at the psychosocial level. In traditional Chinese culture, women with SUI perceive social rejection, social isolation, and internalized shame; these factors prompt them to seek medical treatment [37] and may contribute to worse mental QOL. Hence, psychosocial care is an important issue among this group of women. Healthcare professionals should evaluate and understand the symptoms, relevant experiences, perceptions, and demands of women with SUI. They must provide individualized information and healthcare content to maintain or improve the physical and mental QOL of women with SUI.

Similar to what was proposed, the results of the current study showed that women’s physical state exhibits an important correlation with their physical QOL. For example, women with chronic diseases and poor physical health and those in perimenopause or post-menopause have poor physical QOL. Previous studies have shown that women who are entering menopause [38] or in post-menopause [39] have a higher incidence of urinary incontinence. Moreover, women with urinary incontinence and chronic disease [40] have poorer physical QOL than do women without these health problems. A study on women with urinary incontinence showed that those women with comorbidity and pelvic organ prolapse had worse physical QOL than did those without these health issues [35]. The physical state of women with SUI is closely related to their physical QOL. Therefore, healthcare professionals must conduct more evaluations and focus more attention on women with chronic diseases who are in perimenopause or post-menopause and in a poor health state. These women should be provided with appropriate treatment and care to maintain or improve their physical QOL.

The overall mean positive thinking score of women in the current study was 68.69, which is slightly better than that of patients who are receiving hormone treatment for advanced prostate cancer (66.1) [41] or middle-aged women in the community (66.8) [20]. The reason for such a difference in positive thinking scores among the population remains unknown, and additional research is necessary to clarify this issue. Although women with greater positive thinking experience better physical and mental QOL, positive thinking does not significantly predict urinary incontinence-specific QOL among women in areas such as physical activities, traveling, social/family relationships, and emotional health. No previous studies have explored the correlation between positive thinking and health-related QOL among women with SUI. Research on the general population has shown that people with greater positive thinking have higher life satisfaction [19]. In addition, studies of patients with prostate cancer have found that patients with greater positive thinking experience better prostate cancer-specific QOL, social/family well-being, emotional well-being, and functional well-being [30]. An individual’s positive thinking is correlated with perceived social support, general self-efficacy, and satisfaction with treatment [41]. Additional research is needed to confirm the effect of positive thinking on the health-related QOL of individuals. In future research, women with SUI can be encouraged to maintain adequate positive thinking to improve their physical and mental QOL, including setting and pursuing reasonable life goals and receiving social support from others.

In the current study, women with SUI who experienced less symptom distress of urinary incontinence had better urinary incontinence-specific QOL. In addition, the women with SUI in the current study who had higher self-efficacy for urinary incontinence experienced better health-related QOL, including physical, mental, and urinary incontinence-specific QOL. Such a finding is congruent with the results of previous studies on elderly patients with symptom distress of urinary incontinence. In Başer Seçer et al.’s study, elderly patients were divided into the low group (0–40), the medium group (41–80), and the high group (81–120) in accordance with their GSE-UI scores [17]. Among the three groups, the low group had the worst symptoms of urinary incontinence and the worst urinary incontinence-specific QOL. Healthcare professionals could enhance self-efficacy for urinary incontinence among women with SUI by providing them with relevant information, such as management strategies for SUI, to improve urinary incontinence and health-related QOL.

### Limitations

This study was conducted during the COVID-19 pandemic. Accordingly, patients who visited the hospital during this period might have been caring more about their urinary incontinence symptoms. Hence, the findings of this study cannot be generalized to women with SUI who chose not to seek treatment in a hospital due to various reasons. Further, previous studies have noted that a correlation exists among urinary incontinence symptoms, distress, depression, and health-related QOL [10, 42]. Major depression cases were excluded in the current study to avoid interference to the relationship among research variables. Such an exclusion, however, may cause the overestimation of the health-related QOL of women with SUI. In our future research, women with SUI who suffer from major depression will be included to compare the difference in health-related QOL between women with and without depressive symptoms. A cross-sectional study showed that the correlation among variables was preliminarily elucidated; however, insights into the relevant experiences of women with SUI that change over time could not be obtained. In future research, a combination of longitudinal and qualitative study designs can be used to gain an in-depth and comprehensive understanding of the experiences of women with SUI.

### Recommendations

This study found that a variety of physical and psychosocial factors affect the health-related QOL of women with SUI. Numerous interventions are currently used to improve urinary incontinence symptoms, including PFMT [43, 44] and hybrid telerehabilitation [44]. One study [45] explored the effectiveness of self-management programs for urinary incontinence among elderly women. Self-management programs were determined to be acceptable to women with urinary incontinence, and they could help to improve anxiety, symptom severity, and urinary incontinence symptoms. Although the aforementioned study did not report a statistically significant improvement in the effectiveness of the self-management program in improving QOL and self-efficacy, participants claimed that the intervention made them more confident and motivated to manage their condition. Therefore, to improve health-related QOL among women with SUI, intervention programs should evaluate, manage, and concurrently monitor physical symptoms, symptoms of distress caused by urinary incontinence, and the psychosocial status of patients.

## Conclusions

The results of this study show that women with SUI who have a chronic disease and are in perimenopause or post-menopause and a poor health state have poor physical QOL. Women with SUI who have greater perception of positive thinking experience better general QOL. Women with higher self-efficacy for urinary incontinence have better general QOL and urinary incontinence-specific QOL. Women with fewer symptoms of urinary incontinence experience better urinary incontinence-specific QOL. Healthcare professionals can improve these factors to maintain or improve the health-related QOL of women with SUI.

## Data Availability

The data that support the findings of this study are available upon reasonable request from the first author. The data are not publicly available due to the consideration of ethics, and research data should be used only for academic purposes.

## Abbreviations

COVID-19: Coronavirus Disease 2019
ExMI: Exceptional magnetic innervation
GSE-UI: Geriatric Self-efficacy Index for Urinary Incontinence
IIQ-7: Incontinence Impact Questionnaire Short Form
MCS: Mental component summary
PCS: Physical component summary
PFMT: Pelvic floor muscle training
QOL: Quality of life
SD: Standard deviation
SF-12: 12-Item Short-Form Health Survey
SUI: Stress urinary incontinence
UDI: Urogenital distress inventory
